# Measurement of Sexual Behavior Stigma in Cisgender Mexican Sexual Minority Men: Contextual Considerations of Living in Mexico or the United States

**DOI:** 10.1007/s10508-025-03184-5

**Published:** 2025-07-14

**Authors:** Jane J. Lee, Liying Wang, John Mark Wiginton, Sarah M. Murray, Stefan D. Baral, Roxanne P. Kerani, David A. Katz, Tanmay Bhanushali, Anh-Minh Nguyen, Yingying Wei, Ricardo Baruch-Dominguez, Travis H. Sanchez, Laramie R. Smith

**Affiliations:** 1 Suzanne Dworak-Peck School of Social Work, University of Southern California, 669 West 34th Street, Los Angeles, CA 90089-0411 USA; 2 School of Social Work, University of Washington, Seattle, WA USA; 3 Institute on Digital Health and Innovation, College of Nursing, Florida State University, Tallahassee, FL USA; 4 Center for Population Health and Health Empowerment, College of Nursing, Florida State University, Tallahassee, FL USA; 5 Department of Medicine, University of California San Diego, San Diego, USA; 6 Department of Mental Health, Johns Hopkins University Bloomberg School of Public Health, Baltimore, MD USA; 7 Program for Implementation and Equity Research, Department of Epidemiology, Johns Hopkins University Bloomberg School of Public Health, Baltimore, MD USA; 8 Department of Medicine, University of Washington, Seattle, WA USA; 9 Department of Global Health, University of Washington, Seattle, WA USA; 10 University of Washington, Seattle, USA; 11 Clinica VIHva, Mexico City, Mexico; 12 Department of Epidemiology, Emory University Rollins School of Public Health, Atlanta, GA USA; 13 Division of Infectious Diseases and Global Public Health, University of California San Diego, La Jolla, CA USA

**Keywords:** Stigma, Measurement invariance, Nativity, Sexual orientation, Sexual behavior

## Abstract

We used data from the 2018 and 2019 cycles of the American Men’s Internet Survey (AMIS) and the *Encuesta de Sexo Entre Hombres* (ESEH) (2017) to investigate measurement invariance of a sexual behavior stigma scale among Mexican gay, bisexual, and other sexual minority men (SMM). Employing both a two- and three-group multiple group confirmatory factor analysis (MGCFA), we established configural invariance of the scale, suggesting a uniform factor pattern structure across Mexican SMM subgroups based on country of origin and country of residence: (1) Mexican SMM in the ESEH dataset (*N* = 15,015), (2) Mexican SMM born in Mexico in the AMIS data (*N* = 218), and (3) Mexicans born in the USA in the AMIS data (*N* = 1606). The three-group MGCFA models, which separated the Mexican SMM in the USA who were born in Mexico from those who were born in the USA, demonstrated greater robustness, suggesting that the experiences of stigma among individuals within each group were more similar than when the two AMIS groups were combined. The nuanced findings underscore the importance of considering both nativity and current residence in studying sexual behavior stigma among Mexican SMM. Variations in factor loadings and prevalence of violence-related items across subgroups highlight the contextually dependent nature of sexual behavior stigma and emphasize the need for context-specific measurement tools to comprehensively understand the multifaceted nature of sexual behavior stigma within diverse populations and settings.

**Supplementary Information:**

The online version contains supplementary material available at 10.1007/s10508-025-03184-5.

## Introduction

Gay, bisexual, and other sexual minority men (SMM) disproportionately experience victimization and discrimination due to social stigma related to their sexual behaviors (Altman et al., 2012; Harper & Schneider, 2003). Consequently, sexual behavior stigma, or the devaluation of individuals based on their sexual orientations, identities, or behaviors, has profound impacts on their health and well-being (Layland et al., 2020; Pachankis & Bränström, 2018; Pachankis et al., 2015). Examples of the adverse effects of sexual behavior stigma include avoidance of seeking health care and engagement in HIV-related risk behaviors (Stahlman et al., 2016, 2017). While sexual behavior stigma has been widely acknowledged as a barrier to the uptake of HIV treatment and prevention services among SMM (Babel et al., 2021; Stahlman et al., 2017), gaps remain in our understanding of sexual behavior stigma across social and cultural contexts.

In Mexico, anti-gay attitudes and stigma against SMM may be driven by religiosity and endorsement of gendered roles (Steffens et al., 2015), particularly related to the concept of *machismo*, or male dominance, sexual power, and toughness, which SMM may be perceived as transgressing (Steffens et al., 2015; Wilkerson et al., 2009). In a national survey of lesbian, gay, bisexual, or transgender (LGBT) youth in Mexico, nearly 75 percent of gay and bisexual men reported experiences of homophobic bullying (Baruch-Dominguez et al., 2016). Stigma among SMM in Mexico has been linked to increased sexual risk behaviors (Caballero-Hoyos et al., 2022), feelings of shame and poor mental health (Verduzco, 2016), and lower engagement in HIV prevention behaviors (Calabrese et al., 2018; Kadiamada-Ibarra et al., 2021; Pines et al., 2016). As SMM are among the communities most affected by HIV in Mexico, with an estimated HIV prevalence of 17.4 percent (Algarin et al., 2023), the interplay of sexual behavior stigma and the prevailing heteronormativity within the country have implications for HIV risk and prevention in this population (Caballero-Hoyos et al., 2022).

Considering the influence of sexual behavior stigma on the overall mental health, physical well-being, and utilization of HIV-related services among SMM, there is an increasing focus on investigating stigma-related experiences and developing interventions aimed at alleviating stigma’s effects (Auerbach et al., 2011). However, the cultural subtleties that are characteristic of experiencing stigma add to the complexity of this research within diverse cultural environments, contributing to the lack of consistent measures of stigma across settings (Pescosolido & Martin, 2015). This is particularly notable for validated stigma measures pertinent to SMM living in areas outside of high-income countries (Fitzgerald-Husek et al., 2017).

Further, as migration rates rise, individuals may find opportunities to relocate to countries that may more outwardly embrace diverse sexual identities (United Nations, 2017). In the USA, the country with more immigrants than any other country in the world (Radford, 2019), Mexicans comprise the largest group of foreign-born individuals (Zong & Batalova, 2017). Thus, Mexican SMM in the USA may find themselves in an environment with distinct cultural norms, while still retaining certain values inherent to their country of origin or their ethnic heritage (Egan et al., 2011; Nieves-Lugo et al., 2019). The bidirectional model of acculturation, recognizing the dynamic interaction between the host culture and the culture of origin, further emphasizes that individuals may actively contribute to and shape the cultural landscape of their communities in the USA (Flannery et al., 2001). Additionally, variations in attitudes about SMM within different US communities may add complexity to the experiences of Mexican SMM, highlighting the importance of considering the diverse cultural contexts in which they live. This interaction of cultures and contexts can shape how SMM experience and perceive stigma and thus complicate approaches to measuring this construct.

Notably, sexual behavior stigma has been previously captured by a 13-item sexual behavior stigma scale measuring perceived, anticipated, and enacted stigma among SMM in the USA (Wiginton et al., 2022a), and the same measure has been validated in a national sample of SMM in Mexico (Wiginton et al., 2022b). While studies determined a consistent factor structure of the measure across both contexts (Wiginton et al., 2022a, 2022b), it remains unclear whether the stigma constructs indicated by the factor structure relay the same meaning cross-contextually among Mexican SMM (Putnick & Bornstein, 2016). Thus, we sought to examine the measurement invariance of the 13-item sexual behavior stigma scale among Mexican SMM in the USA and in Mexico. Specifically, we assessed measurement invariance of the sexual behavior stigma scale across SMM who (1) were born in Mexico and are living in the U.S., (2) are of Mexican heritage and were born in the USA and are living in the USA, and (3) were born and are living in Mexico.

## Method

### Participants

We used data from the 2018 and 2019 cycles of the American Men’s Internet Survey (AMIS) and the *Encuesta de Sexo Entre Hombres* (ESEH) (2017), both of which are web-based national, cross-sectional surveys with the aim to assess sexual behaviors, sexual health, and experiences of stigma of cisgender men who have sex with men in the USA and Mexico, respectively (Baruch-Domínguez et al., 2022; Emory University, 2017). AMIS collects data annually in the USA with an aim to recruit approximately 10,000 participants each year through convenience sampling using web-based recruitment strategies such as social networking websites, banner ads, email blasts, and dating apps. Eligible participants are at least 15 years of age, report being assigned male sex at birth and current male gender identity (i.e., are cisgender men), reside in the USA, and report oral or anal sex with a male partner at least once in the past. ESEH used similar methods to collect data from SMM in Mexico in 2017 who were at least 18 years of age, identified as a cisgender man, resided in Mexico, and reported oral or anal sex with another man. ESEH participants were recruited via social networking applications, such as Facebook, Twitter, Grindr, and Hornet.

### Procedure

All participants provided informed consent and completed their respective survey on secure servers administered by SurveyGizmo (now Alchemer) (Boulder, CO, USA).

### Measures

We assessed participants’ age, education, sexual identity, and living situation (experiencing homeless or not). We also examined participants’ self-reported prior HIV testing behaviors and HIV status.

Sexual behavior stigma was assessed in both surveys via a set of the same 13 items that represented perceived, enacted, and anticipated stigma. As previously noted, the scale has been previously validated in multiple large samples of MSM (Wiginton et al., 2022a, 2022b). For the ESEH study, there were five response options for each item (yes, no, not applicable, prefer not to answer, I don’t know). For the AMIS study, affirmative responses included “Yes, in the past 6 months” and “Yes, but not in the past 6 months.” These two responses were collapsed into as a single “Yes” response for the purpose of this analysis. For both studies, only “Yes” and “No” responses were considered for analysis, with other responses treated as missing. Only complete cases were included in our analysis.

Two of the thirteen items were assessed via a two-step process. Enacted stigma in the form of sexual violence was assessed by asking whether the participant had experienced sexual violence and was subsequently asked whether they believed the sexual violence was related to their having sex with men. Endorsement of both the experience of sexual violence and the belief that it was related to having sex with men was coded as an affirmative response, while no experience or experience without attribution to sexual behavior was coded as “No.” The items assessing physical violence were assessed in the same manner.

### Statistical Analysis

Our analyses included ESEH participants and AMIS participants who identified as Mexican. Analyses were focused on three groups: (1) AMIS participants who were born in Mexico but living in the USA; (2) AMIS participants who were born in the USA and reported being Mexican; and (3) ESEH participants (i.e. individuals living in Mexico). Appropriate tests (ANOVA and chi-square) were used to compare demographic characteristics across groups. Pearson's chi-square tests were also conducted to test for differences in the distribution of each endorsed item across the three groups.

We performed a confirmatory factor analysis (CFA) on the AMIS data and the ESEH data independently to establish the fit of the three-factor model that was identified in prior analyses, which included (1) stigma from family and friends (Items 1–3), (2) anticipated healthcare stigma (Items 4–5), and (3) general social stigma (Items 6–13) (Augustinavicius et al., 2020). We used a weighted least squares estimator with robust standard errors and a mean- and variance adjusted test statistic (WLSMV) to conduct the CFA, which is more suitable for categorical and ordinal data, using the *lavaan* package in R (Hirschfeld & von Brachel, 2014). The variances of the latent variables were fixed to 1. The following statistics were used to assess adequate model fit for evaluating configural invariance: Root Mean Square Error of Approximation (RMSEA) < 0.05; Comparative Fit Index (CFI) > 0.90; Tucker Lewis Index (TLI) > 0.90; and the Standardized Root Mean Square Residual (SRMR) < 0.08.

We also conducted two-group and measurement invariance testing to test whether the same factor structure and factor loadings were shared between (1) Mexican SMM in the ESEH data and (2) Mexican SMM in the AMIS data, as well as three group measurement invariance testing to compare measurement among: (1) Mexican SMM in the ESEH dataset, (2) Mexican SMM born in Mexico in the AMIS data, and (3) Mexicans born in the USA in the AMIS data.

We used a three-step process to examine measurement invariance and ensure that the latent constructs were comparable across groups while providing insights into the structure of the model. First, we fit a configural model, where no constraints were imposed on the parameters across groups to determine whether the overall structure framework was similar between groups. Subsequently, we implemented a metric model, constraining the factor loadings to be equal between the groups. This step aimed to assess whether the relationships between the observed variables and latent constructs were equivalent across groups. The fit of the metric model was evaluated using standard fit indices (change in CFI and change in RMSEA) and the significance of the chi-square test. Specifically, a decrease in CFI greater than 0.01 or an increase in RMSEA greater than 0.015 between the configural and metric models was indication of potential issues with metric invariance. In case of a significant chi-square test result, indicating lack of metric invariance, further steps, such as partial metric invariance testing, were explored to identify specific parameters contributing to the lack of invariance. Specifically, we selectively freed factor loadings based on the Score test (Lagrange Multiplier test) for releasing constrained parameters in the model (van de Schoot et al., 2012). The selection of items for release followed the order of statistical significance obtained through the Score test, starting with the most significant item (van de Schoot et al., 2012). We started with the most significant item and successively freed factor loadings and compared models until partial invariance was achieved. We then evaluated scalar invariance, which involved constraining intercepts in addition to factor loadings. For scalar invariance, we considered similar fit indices and criteria as in metric invariance as well as the change in intercepts. A CFI greater than 0.01, a RMSEA greater than 0.015, and significant chi-square were indications of issues with scalar invariance. All analyses were conducted in R version 4.1.3 (Hirschfeld & von Brachel, 2014).

## Results

After removing duplicated AMIS survey responses across years (2018–2019), there were 1824 AMIS participants who identified as Mexican. Among these participants, 218 were born in Mexico and 1606 were born in the USA. Within the ESEH dataset, there were a total of 15,875 participants, 860 of whom did not complete the survey, leaving 15,015 participants in the analytic sample. Table 1 presents the demographic characteristics of participants in each of these three groups. The mean age of the participants across the groups was 27.9 years (SD = 8.4), with two thirds less than 30 years old. More than half of the participants (61.2%) obtained bachelor’s degrees or higher. The majority of participants identified as gay (74.9%) and approximately 15.7 percent identified as bisexual. Only 104 (0.9%) participants reported experiencing homelessness. Most study participants (71.9%) had ever tested for HIV. Overall, 10.2% of participants reported being HIV positive.

**Table 1:** Demographic characteristics of Mexican sexual minority men across groups, AMIS (2018–2019) born in Mexico or born in the USA, ESEH (2017)

	AMIS: Born in Mexico (N = 218)	AMIS: Born in the USA (N = 1606)	ESEH (N = 15,015)	Total (N = 16,839)	*p *value
*Age (in years)*					< 0.001^a^
Mean (SD)	27.9 (10.0)	25.1 (9.6)	28.2 (8.1)	27.9 (8.4)	
Range	15.0–80.0	15.0–69.0	18.0–78.0	15.0–80.0	
*Age (categories)*					< 0.001^b^
18–24	108 (49.5%)	1014 (63.1%)	5865 (39.1%)	6987 (41.5%)	
25–29	43 (19.7%)	293 (18.2%)	3947 (26.3%)	4283 (25.4%)	
30–39	39 (17.9%)	161 (10.0%)	3740 (24.9%)	3940 (23.4%)	
40+	28 (12.8%)	138 (8.6%)	1463 (9.7%)	1629 (9.7%)	
*Education*					< 0.001^b^
Secondary or less	16 (7.4%)	200 (12.7%)	360 (2.4%)	576 (3.5%)	
High school	38 (17.7%)	368 (23.3%)	3298 (22.3%)	3704 (22.4%)	
Technical	82 (38.1%)	609 (38.5%)	1449 (9.8%)	2140 (12.9%)	
Bachelor’s or above	79 (36.7%)	403 (25.5%)	9652 (65.4%)	10,134 (61.2%)	
Missing/Unknown	3	26	256	285	
*Sexual identity*					< 0.001^b^
Gay	3 (2.0%)	24 (2.1%)	8744 (84.1%)	8771 (74.9%)	
Bisexual	34 (22.2%)	271 (23.4%)	1531 (14.7%)	1836 (15.7%)	
Heterosexual	116 (75.8%)	859 (74.1%)	50 (0.5%)	1025 (8.8%)	
Prefer not to answer	0 (0.0%)	2 (0.2%)	28 (0.3%)	30 (0.3%)	
Don’t know	0 (0.0%)	3 (0.3%)	42 (0.4%)	45 (0.4%)	
Missing/Unknown	1	11	6	18	
*Homeless*					
No	137 (95.8%)	1062 (95.3%)	10,352 (99.5%)	11,551 (99.1%)	
Yes	5 (3.5%)	50 (4.5%)	49 (0.5%)	104 (0.9%)	
Prefer not to answer	1 (0.7%)	2 (0.2%)	0 (0.0%)	3 (0.0%)	
Don’t know	0 (0.0%)	0 (0.0%)	0 (0.0%)	0 (0.0%)	
Missing/Unknown	11	56	0	67	
*Ever tested for HIV*					
No	36 (23.4%)	366 (31.3%)	2819 (27.6%)	3221 (27.9%)	
Yes	114 (74.0%)	801 (68.5%)	7393 (72.3%)	8308 (71.9%)	
Prefer not to answer	2 (1.3%)	1 (0.1%)	11 (0.1%)	14 (0.1%)	
Don’t know	2 (1.3%)	1 (0.1%)	9 (0.1%)	12 (0.1%)	
Missing/Unknown	0	1	169	170	
*HIV status*					< 0.001^b^
Negative	106 (68.8%)	743 (63.5%)	5937 (57.1%)	6786 (57.9%)	
Positive	4 (2.6%)	49 (4.2%)	1138 (10.9%)	1191 (10.2%)	
Don’t know	44 (28.6%)	378 (32.3%)	3326 (32.0%)	3748 (32.0%)	

^a^Linear model ANOVA

^b^*χ*^2^ test

For the measurement invariance testing of the sexual behavior sigma items, we removed observations with missing values. This resulted in 1324 observations in the AMIS data with 154 Mexican SMM born in Mexico and 1170 Mexican SMM born in the USA. Within the ESEH dataset, 10,401 observations were included for our analyses after removing observations with missing values. Supplementary Table 1 provides the demographic characteristics of the complete cases of Mexican SMM (*N* = 11,725) across the three groups.

Table 2 presents all stigma items and the proportion of participants that endorsed each sexual behavior stigma item across the three groups. Among Mexican SMM born in the USA and living in the USA (Mexican-identifying AMIS participants), the most-endorsed stigma experience was discriminatory remarks/gossip by one’s family (*n* = 638; 48.2%), followed by verbal harassment (*n* = 511*;* 38.6%), and feeling scared to be in public places (*n* = 457; 34.5%). The least-endorsed stigma item was sexual violence (*n* = 26; 2.0%), followed by physical violence (*n* = 37; 2.8%), and having felt mistreated in a health center (*n* = 48; 3.6%). Similarly, among Mexican SMM living in Mexico (ESEH participants), the most-endorsed stigma experience was verbal harassment (*n* = 4,895; 47.1%), followed by discriminatory remarks/gossip by one’s family (*n* = 4543; 43.7%), and feeling scared to be in public places (*n* = 2476; 23.8%). The least-endorsed stigma item was sexual violence (*n* = 742; 7.1%), followed by having felt mistreated in a health center (*n* = 863; 8.3%), and having been gossiped about by healthcare providers (*n* = 884; 8.5%).

**Table 2:** Endorsement of sexual behavior stigma items among Mexican sexual minority men across groups, AMIS (2018–2019) born in Mexico or born in the USA, ESEH (2017)

Item (stigma type)	AMIS: Born in Mexico, N = 154 N (%)	AMIS, Born in U.S N = 1170 N (%)	ESEH N = 10,401 N (%)	Overall, N = 11,725 N (%)	*p*-value^a^
1. Have you ever felt excluded from family activities because you have sex with men? (perceived)					
No	112 (72.7%)	841 (71.9%)	8062 (77.5%)	9015 (76.9%)	< 0.001
Yes	42 (27.3%)	329 (28.1%)	2339 (22.5%)	2710 (23.1%)	
2. Have you ever felt that family members have made discriminatory remarks or gossiped about you because you have sex with men? (perceived)					
No	81 (52.6%)	605 (51.7%)	5858 (56.3%)	6544 (55.8%)	0.008
Yes	73 (47.4%)	565 (48.3%)	4543 (43.7%)	5181 (44.2%)	
3. Have you ever felt rejected by your friends because you have sex with men? (perceived)					
No	121 (78.6%)	931 (79.6%)	8373 (80.5%)	9425 (80.4%)	0.600
Yes	33 (21.4%)	239 (20.4%)	2028 (19.5%)	2300 (19.6%)	
4. Have you ever felt afraid to go to healthcare services because you worry someone may learn you have sex with men? (anticipated)					
No	115 (74.7%)	865 (73.9%)	8362 (80.4%)	9342 (79.7%)	< 0.001
Yes	39 (25.3%)	305 (26.1%)	2039 (19.6%)	2383 (20.3%)	
5. Have you ever avoided going to healthcare services because you worry someone may learn you have sex with men? (anticipated)					
No	123 (79.9%)	926 (79.1%)	8997 (86.5%)	10,046 (85.7%)	< 0.001
Yes	31 (20.1%)	244 (20.9%)	1404 (13.5%)	1679 (14.3%)	
6. Have you ever felt that you were not treated well in a health center because someone knew that you have sex with men? (perceived)					
No	146 (94.8%)	1130 (96.6%)	9538 (91.7%)	10,814 (92.2%)	< 0.001
Yes	8 (5.2%)	40 (3.4%)	863 (8.3%)	911 (7.8%)	
7. Have you ever heard healthcare providers gossiping about you (talking about you) because you have sex with men? (enacted)					
No	141 (91.6%)	1093 (93.4%)	9517 (91.5%)	10,751 (91.7%)	0.079
Yes	13 (8.4%)	77 (6.6%)	884 (8.5%)	1974 (8.3%)	
8. Have you ever felt that the police refused to protect you because you have sex with men? (perceived)					
No	142 (92.2%)	1090 (93.2%)	8680 (83.5%)	9912 (84.5%)	< 0.001
Yes	12 (7.8%)	80 (6.8%)	1721 (16.5%)	1813 (15.5%)	
9. Have you ever felt scared to be in public places because you have sex with men? (perceived)					
No	106 (68.8%)	761 (65.0%)	7925 (76.2%)	8792 (75.0%)	< 0.001
Yes	48 (31.2%)	409 (35.0%)	2476 (23.8%)	2933 (25.0%)	
10. Have you ever been verbally harassed and felt it was because you have sex with men? (enacted)					
No	103 (66.9%)	710 (60.7%)	5506 (52.9%)	6319 (53.9%)	< 0.001
Yes	51 (33.1%)	460 (39.3%)	4895 (47.1%)	5406 (46.1%)	
11. Have you ever been blackmailed by someone because you have sex with men? (enacted)					
No	140 (90.9%)	1014 (86.7%)	8657 (83.2%)	9811 (83.7%)	< 0.001
Yes	14 (9.1%)	156 (13.3%)	1744 (16.8%)	1914 (16.3%)	
12. Has someone ever physically hurt you (pushed, shoved, slapped, hit, kicked, choked or otherwise physically hurt you)? [AND] Do you believe any of these experiences of physical violence was/were related to the fact that you have sex with men? (enacted)					
No	150 (97.4%)	1137 (97.2%)	8829 (84.9%)	10,116 (86.3%)	< 0.001
Yes	4 (2.6%)	33 (2.8%)	1572 (15.1%)	1609 (13.7)%	
13. Have you ever been forced to have sex when you did not want to (by forced, I mean physically forced, coerced to have sex, or penetrated with an object, when you did not want to)? [AND] Do you believe any of these experiences of sexual violence were related to the fact that you have sex with men? (enacted)					
No	152 (98.7%)	1146 (97.9%)	9659 (92.9%)	10,957 (93.4%)	< 0.001
Yes	2 (1.3%)	24 (2.1%)	742 (7.1%)	768 (6.6%)	

^a^*χ*^2^ test

The proportion of participants endorsing items (10 out of 13) differed across three groups (Table 2). However, there were no significant differences in stigma endorsement between Mexican SMM born in the USA and Mexican SMM born in Mexico residing in the USA (AMIS; Table 3). The significant difference in the three-group chi-square test was driven by the ESEH participants (Mexican SMM residing in Mexico). The two groups (Mexican SMM born in Mexico living in the USA and Mexican SMM born in the USA living in the USA) from the AMIS dataset consistently reported higher prevalence of several perceived and anticipated stigma items (e.g., exclusion from family activities) and lower prevalence of enacted stigma items (e.g., physical violence due to sexual behavior as MSM and sexual violence due to sexual behavior as MSM), compared to those living in Mexico (ESEH participants).

**Table 3:** Endorsement of sexual behavior stigma items among Mexican sexual minority men, AMIS (2018–2019) born in Mexico and born in the U.S

Item (stigma type)	United States, N = 1170	Mexico, N = 154	Overall, N = 1324	*p* value^a^
1. Have you ever felt excluded from family activities because you have sex with men? (perceived)				
No	841 (71.9%)	112 (72.7%)	953 (72.0%)	0.826
Yes	329 (28.1%)	42 (27.3%)	371 (28.0%)	
2. Have you ever felt that family members have made discriminatory remarks or gossiped about you because you have sex with men? (perceived)				
No	605 (51.7%)	81 (52.6%)	686 (51.8%)	0.836
Yes	565 (48.3%)	73 (47.4%)	638 (48.2%)	
3. Have you ever felt rejected by your friends because you have sex with men? (perceived)				
No	931 (79.6%)	121 (78.6%)	1052 (79.5%)	0.773
Yes	239 (20.4%)	33 (21.4%)	272 (20.5%)	
4. Have you ever felt afraid to go to healthcare services because you worry someone may learn you have sex with men? (anticipated)				
No	865 (73.9%)	115 (74.7%)	980 (74.0%)	0.843
Yes	305 (26.1%)	39 (25.3%)	344 (26.0%)	
5. Have you ever avoided going to healthcare services because you worry someone may learn you have sex with men? (anticipated)				
No	926 (79.1%)	123 (79.9%)	1049 (79.2%)	0.835
Yes	244 (20.9%)	31 (20.1%)	275 (20.8%)	
6. Have you ever felt that you were not treated well in a health center because someone knew that you have sex with men? (perceived)				
No	1130 (96.6%)	146 (94.8%)	1276 (96.4%)	0.268
Yes	40 (3.4%)	8 (5.2%)	48 (3.6%)	
7. Have you ever heard healthcare providers gossiping about you (talking about you) because you have sex with men? (enacted)				
No	1093 (93.4%)	141 (91.6%)	1234 (93.2%)	0.389
Yes	77 (6.6%)	13 (8.4%)	90 (6.8%)	
8. Have you ever felt that the police refused to protect you because you have sex with men? (perceived)				
No	1090 (93.2%)	142 (92.2%)	1232 (93.1%)	0.661
Yes	80 (6.8%)	12 (7.8%)	92 (6.9%)	
9. Have you ever felt scared to be in public places because you have sex with men? (perceived)				
No	761 (65.0%)	106 (68.8%)	867 (65.5%)	0.353
Yes	409 (35.0%)	48 (31.2%)	457 (34.5%)	
10. Have you ever been verbally harassed and felt it was because you have sex with men? (enacted)				
No	710 (60.7%)	103 (66.9%)	813 (61.4%)	0.137
Yes	460 (39.3%)	51 (33.1%)	511 (38.6%)	
11. Have you ever been blackmailed by someone because you have sex with men? (enacted)				
No	1014 (86.7%)	140 (90.9%)	1154 (87.2%)	0.139
Yes	156 (13.3%)	14 (9.1%)	170 (12.8%)	
12. Has someone ever physically hurt you (pushed, shoved, slapped, hit, kicked, choked or otherwise physically hurt you)? [AND] Do you believe any of these experiences of physical violence was/were related to the fact that you have sex with men? (enacted)				
No	1137 (97.2%)	150 (97.4%)	1287 (97.2%)	> 0.999
Yes	33 (2.8%)	4 (2.6%)	37 (2.8%)	
13. Have you ever been forced to have sex when you did not want to (by forced, I mean physically forced, coerced to have sex, or penetrated with an object, when you did not want to)? [AND] Do you believe any of these experiences of sexual violence were related to the fact that you have sex with men? (enacted)				
No		152 (98.7%)	1298 (98.0%)	0.760
Yes		2 (1.3%)	26 (2.0%)	

^a^*χ*^2^ test

Table 4 presents the fit statistics from the measurement invariance testing through the multigroup CFA of the two-group model: ESEH data (Mexican SMM in Mexico) and AMIS data (Mexicans in the USA regardless of country of birth). The two group configural model with no constraints indicated good fit. Next, a metric model was implemented with factor loadings constrained to be equal between the two groups. However, with Δ*χ*^2^ $=$ 94.92 and *p* < .001, a significant chi-square test, we inferred a lack of metric invariance between stigma items in the two groups. Thus, a partial metric invariance model was attempted in which factor loadings for stigma Items 12, 13, 8, 6, 11, and 3 were freed (i.e., not constrained to equality across group) based on the Score test (Lagrange Multiplier test) for releasing one or more fixed or constrained parameters in the model (van de Schoot et al., 2012). The order of these items indicates the order of statistical significance when running the score test. We first released the most significant item and then compared the two models again until we reached partial invariance. With a new nonsignificant chi-square test of Δ*χ*^2^ = 7.54 and *p* < .11, a partial metric invariance was established for the two groups. The adjusted metric model also demonstrated better fit, with an RMSEA of 0.037, CFI of 0.97, and TLI of 0.963 versus the metric model which had an RMSEA of 0.046, CFI of 0.951, and TLI of 0.943.

**Table 4:** Measurement invariance testing, AMIS and ESEH datasets (Two groups*)

Model	*χ*^2^	*df*	*p* value	RMSEA	90% CI lower	90% CI upper	CFI	TLI	SRMR
AMIS model	180.679	62	< 0.001	0.038	0.032	0.045	0.967	0.959	0.062
ESEH model	947.571	62	< 0.001	0.037	0.035	0.039	0.971	0.963	0.043
Configural model	1128.25	124	< 0.001	0.037	0.035	0.039	0.97	0.963	0.043
Metric model	1783.978	134	< 0.001	0.046	0.044	0.048	0.951	0.943	0.047
Adjusted Metric model	1150.755	128	< 0.001	0.037	0.035	0.039	0.97	0.963	0.043
Adjusted Scalar model	1157.723	133	< 0.001	0.036	0.034	0.038	0.97	0.965	0.043

*Two groups included Mexican SMM from the ESEH data and Mexican SMM from the AMIS data

A partial scalar invariance was also attempted with the same adjustments made for the partial metric invariance. Additional adjustment was needed due to a significant chi-square test. Intercepts of stigma Items 8, 9, 12, and 13 along with the loading parameter of Item 9 were also adjusted to establish a partial scalar invariance between the two groups. Again, these items were adjusted based on the Score test (Lagrange Multiplier test) for releasing fixed or constrained parameters in the model. This is evidenced by a nonsignificant chi-square test of Δ*χ* = 6.89 and *p* < .22. We established a 2-group configural, partial metric, and partial scalar invariance model for Mexican SSM in the USA and Mexican SMM in Mexico (ESEH participants).

Fit statistics for the models, estimated separately for the three groups: (1) Mexican SMM in the ESEH dataset, (2) Mexican SMM born in Mexico in the AMIS data, and (3) Mexicans born in the USA in the AMIS data, are presented in Table 5, and demonstrated good fit. The configural model with the three-group CFA with no constraints on factor loadings showed good fit to the data. The metric model with factor loadings constrained to be equal across three groups demonstrated relatively worse fit compared to the configural model (Δ*χ*^2^ = 54.88, Δ*df* = 20, *p* < .001), and metric invariance was not established. We adjusted the loading parameters of four items (Items 9, 10, 12, and 13) (based on Lagrange Multiplier test) and established partial metric invariance: Δ*χ*^2^ = 16.67, Δ*df* = 12, *p* = .16. Similarly, partial scalar invariance was established with adjustments for the intercepts of Items 9 and 12 (based on Lagrange Multiplier test), in addition to the adjustments made to the loading parameters in the adjusted metric model (Δ*χ*^2^ = 15.1, Δ*df* = 14, *p* = .37). All adjustments were made on the items that were loaded on factor 3 (general stigma) (Supplementary Table 2). Items in Factor 1 (stigma from family and friends) and Factor 2 (anticipated healthcare stigma) were comparable across the three groups.

**Table 5:** Measurement invariance testing, AMIS and ESEH datasets (Three groups*)

Model	*χ*^2^	*df*	*p* value	RMSEA	90% CI lower	90% CI upper	CFI	TLI	SRMR
ESEH model	947.571	62	< 0.001	0.037	0.035	0.039	0.971	0.963	0.043
AMIS US model	164.694	62	< 0.001	0.038	0.031	0.045	0.967	0.958	0.063
AMIS Mexico model	56.933	62	0.658	0	0	0.041	1.000	1.013	0.092
Configural model	1169.198	186	< 0.001	0.037	0.035	0.039	0.971	0.964	0.043
Metric model	1941.455	206	< 0.001	0.046	0.045	0.048	0.949	0.942	0.048
Adjusted Metric model	1277.873	198	< 0.001	0.037	0.035	0.039	0.968	0.962	0.044
Adjusted Scalar model	1293.486	212	< 0.001	0.036	0.034	0.038	0.968	0.965	0.044

*Three groups included (1) Mexican SMM from ESEH, (2) Mexican SMM born in the USA from AMIS, (3) Mexican SMM born in Mexico from AMIS

## Discussion

The current study used multiple group CFA (MGCFA) to examine measurement invariance of a sexual behavior stigma scale among groups of Mexican SMM participants from AMIS and ESEH. We used two approaches to subset the data for assessing measurement invariance: the first categorized the samples into two groups (Mexican SMM in Mexico vs. Mexican SMM living in the US); the second categorized the samples into three groups (Mexican SMM in Mexico, Mexican SMM born in Mexico in the USA, Mexican SMM born in the USA and in the USA). The 3-factor structure of the sexual behavior scale identified in prior studies with the same items and with the same AMIS and ESEH data (Augustinavicius et al., 2020; Wiginton et al., 2022a, 2022b) was replicated in these samples, demonstrating adequate fit in all subgroups. As this model indicated fit across all subgroups of the same latent structure, we determined that there was configural invariance across the three subgroups of Mexican SMM. This implies that the sexual behavior stigma scale used in this study is measuring the same underlying construct across these diverse subgroups. Notably, the observed partial metric and scalar invariance indicate that, despite some variations in the factor loadings and intercepts for specific items, the scale maintains a consistent measurement metric and scale across these groups.

Partial metric and scalar invariance were also established across the subgroups after releasing factor loading and intercept constraints for several items. The observed partial metric and scalar invariance indicate that, despite some variations in the factor loadings and intercepts for specific items, the scale maintains a consistent measurement metric and scale across these groups. While the partial metric and scalar models had similar fit in both the two-group MGCFA and three-group MGCFA, the three-group MGCFA models were more robust based on indicators of robustness. This suggests that by distinguishing between Mexican SMM in the USA who were born in Mexico and those who were born in the USA, the perceptions and experiences of stigma within each group were more comparable than when both AMIS groups were combined. It is important to note that in the measurement of these constructs, the similarity lies in the relationship between the indicators and the underlying constructs, rather than in the specific level or magnitude of stigma endorsed by individuals within each group.

The findings from our study have important implications for researchers working in the field of sexual behavior stigma. The establishment of configural invariance suggests that the same factor pattern structure applies across Mexican SMM in Mexico, those born in Mexico living in the USA, and those born in the USA and living in the USA. Researchers should consider these nuances when comparing the levels of sexual behavior stigma among Mexican SMM across different subgroups. The robustness of the three-group MGCFA models suggests that separating Mexican SMM born in Mexico in the USA from those born in the USA provides a more nuanced understanding of stigma experiences within each group, highlighting the importance of considering both nativity and current residence in future research and intervention efforts targeting sexual behavior stigma in this population.

As constraints were released for seven items in the two-group MGCFA and four items in the three-group MGCFA to establish partial metric and scalar invariance, non-invariance was not trivial. All but one item adjusted in the MGCFA loaded onto Factor 3 (general sexual stigma). Therefore, the items in factor 3 may be the driver of non-invariance in the measure. The lack of metric and scalar invariance suggests that the items measuring general sexual stigma may not be applicable across all three subgroups of Mexican SMM (van de Schoot et al., 2012). A closer examination of the items that loaded onto factor 3 revealed that five of the eight items aimed to measure enacted stigma. Hence, the experience of enacted sexual behavior stigma may be highly context-dependent, with greater relevance to the concept of stigma in one cultural or sociopolitical context versus another (Zahn et al., 2016).

Notably, the factor loadings of Item 11 (blackmail), 12 (physical violence), and 13 (forced sex) were the highest among Mexican SMM in the ESEH dataset and the subset of Mexican SMM in the AMIS dataset who were born in Mexico. The prevalence of these same items was also highest in the ESEH dataset, compared to the prevalence among the two subgroups within the AMIS dataset (Table 3). Similarly, a study examining sexual behavior stigma between countries in western Africa and the USA found higher rates of blackmailing in the African sample, compared to the USA (Stahlman et al., 2016). Taken together, these findings suggest that some experiences of enacted sexual behavior stigma, as well as the extent to which such experiences tap into the broader construct of sexual behavior stigma, may vary based on contextual influences.

Our finding that there were differences in the factor loadings and the prevalence of violence items across the three subgroups of Mexican SMM may be the result of the contextual-dependent nature of enacted stigma. Sexual behavior stigma may be more likely to manifest as violence in a context where social protections are not well established (Duarte-Gómez et al., 2018; UNICEF, n.d.). This aligns with our finding that the prevalence of violence linked to sexual behavior stigma was similar between the two subgroups of Mexican SMM in the AMIS dataset who reside in the USA and may experience similar levels of social protections. Further, greater tolerance of violence in Mexico may explain the difference in factor loadings across the three subgroups (Aguirre Ochoa & Barbosa Muñoz, 2013). Certain communities may be more tolerant of physical violence due to greater exposure or accepted manifestations or performance of masculinities, which can contribute to greater reports of enacted stigma in the form of physical and sexual violence (White & Satyen, 2015).

While Mexico is often considered among the more inclusive countries for sexual minorities among low-and-middle-income countries (Lamontagne et al., 2018), SMM still report greater experiences of violence and other forms of discrimination (Wiginton et al., 2022b). Prior studies have indicated that the prevalence of violence related to sexual orientation experienced by SMM in Mexico was as high as 26 percent (Betron & Gonzalez-Figueroa, 2009). The actual prevalence of violence may be higher considering that cultural factors such as *machismo* can lead to underreporting of victimization events that threaten men’s masculinity (Nieves-Rosa et al., 2000). Thus, our findings highlight the potentially greater relevance and prevalence of enacted stigma among Mexican SMM living in Mexico relative to Mexican SMM living in the USA.

Our results point to potential recommendations for future use of the 13-item sexual behavior stigma scale examined in this study. The lack of specificity in general social stigma items, particularly the absence of specified actors, suggests an avenue for refining the scale. Future qualitative research could focus on exploring how the absence of specified actors in these items contributes to variations in interpretation and perception of stigma. Understanding the role of unspecified actors in shaping stigma experiences could lead to the development of more contextually relevant and precise measurement items. Additionally, investigating the impact of actor specification in the context of diverse cultural norms may provide further insights into the sources and dynamics of stigma within specific subgroups.

Another critical consideration for future research is the examination of language and translation issues. Clarifying the language in which the survey was completed by AMIS participants may address potential language-related biases. This information is vital for ensuring the cross-cultural validity of the stigma scale. Research exploring the impact of language differences on item interpretation and responses may facilitate the refinement of the scale for more accurate and culturally sensitive assessments.

Our recommendations for future use of the sexual behavior stigma scale involve a multifaceted approach: Qualitative research may help to untangle the cultural intricacies influencing stigma among Mexican MSM. Refinement of the scale by specifying actors in general social stigma items and investigation of potential language-related biases may enhance the scale's relevance, validity, and applicability across diverse subgroups of Mexican SMM. Expansion of the anticipated stigma subscale is also recommended. Specifically, the subscale would benefit from the addition of one or more items to improve its stability and reliability, as typically three or more items per subscale are recommended for stable latent factors. Further validation studies across different regions of Mexico and various subgroups of SMM (e.g., by age, socioeconomic status, or urban/rural residence) could provide insights into the scale's generalizability and potential need for subgroup-specific adaptations. These efforts may ultimately contribute to more effective interventions and accurate assessments of sexual behavior stigma within this population.

There are several study limitations to consider. The study findings are based on specific samples of Mexican SMM from the AMIS and ESEH datasets, which included participants who were recruited via web-based recruitment methods. This may limit the generalizability of our results to other populations, both within Mexico and Mexican SMM living in other countries. While our study highlights important contextual differences in the manifestation of sexual behavior stigma, cultural norms and social attitudes are complex and multifaceted. Additionally, we did not assess potential covariates such as religious identity, region, acculturation, outness, or socioeconomic status, which could influence how individuals experience and report stigma. For Mexican SMM who migrated to the USA, their reasons for migration as well as their age at migration may also shape their stigma experiences (Lee et al., 2024). Future research should explore additional factors that may contribute to differences in experiences of stigma among Mexican SMM as well as the impacts of stigma across various outcomes in diverse settings. Notably, Supplementary Table 3, which compares the demographic characteristics of complete cases with removed cases due to missing data, revealed that participants who were excluded were more likely to identify as bisexual or 'don't know' and be unaware of their HIV status. While it is reasonable to expect differences as social and demographic characteristics may influence whether individuals are inclined to disclose experiences related to sexual behavior stigma, this demographic divergence highlights the need for careful interpretation of findings and tailored engagement strategies in future studies to better include those less represented in research on stigmatized topics.

Moreover, Supplementary Table 4 indicated that participants with missing data reported higher rates of stigma-related experiences, which suggests that individuals experiencing more severe stigma may have been less likely to complete the stigma items. Across all items in our scale, the average percentage of missing data was 21.49%. The item with the highest percentage of missing data was "Police refused to protect" at 44.97%, while the item with the lowest was "Physically hurt" at 4.99%. This pattern of missing responses highlights potential biases in our findings and emphasizes the need for developing survey methods that better accommodate and encourage full participation from those who may be most affected by stigma. Nevertheless, our comparative analysis showed that WLSMV with pairwise deletion and WLSMV with listwise deletion yielded structurally equivalent results in our case, enhancing our confidence in the robustness of our findings (Supplementary Tables 5.1, 5.2).

Despite these limitations, this study offers valuable insights into the measurement invariance of a sexual behavior stigma scale among different groups of Mexican SMM in the USA and Mexico. Findings underscore the nuanced nature of stigma experiences within this population, highlighting the importance of considering contextual factors in stigma research. Notably, the prevalence of enacted stigma, particularly in the form of violence, varies significantly across different contexts, shedding light on the complex interplay between cultural norms, social protections, and experiences of stigma. These results have implications for refining the measurement tool, suggesting the need for a modified scale that separates violence-related items and addresses potential ambiguities. While this study contributes to our understanding of sexual behavior stigma among Mexican SMM, it also underscores the need for future research and interventions to explore and address additional factors influencing stigma experiences and their broader impact on diverse populations in various settings.

## Supplementary Information

Supplementary file 1

Supplementary file 2

Supplementary file 3

Supplementary file 4

Supplementary file 5

## Data Availability

Requests to access the data from the USA and Mexico can be sent to T. H. Sanchez and L. R. Smith, respectively.

## Code Availability

The code used for the analyses presented in this manuscript is available upon request from the corresponding author.
