# Supplementary material for: Measurement of Sexual Behavior Stigma in Cisgender Mexican Sexual Minority Men: Contextual Considerations of Living in Mexico or the United States
Source: Arch Sex Behav. 2025 Jul 14;54(7):2599–610. doi: 10.1007/s10508-025-03184-5 (PMC12457474; doi:10.1007/s10508-025-03184-5)
Supplement: Supplementary file 1 [file 10508_2025_3184_MOESM1_ESM.docx]

**Supplementary Table 1.** Demographic characteristics of Mexican SMM across groups, AMIS (2018-2019) Born in Mexico or Born in the U.S., ESEH (2017): Complete cases

|  | AMIS: Born in Mexico  (N=154) | AMIS: Born in U.S.  (N=1,170) | ESEH (N=10,401) | Total (N=11,725) | p value |
| --- | --- | --- | --- | --- | --- |
| Age |  |  |  |  | < 0.001^a^ |
| Mean (SD) | 27.7 (10.2) | 25.3 (9.6) | 28.1 (8.0) | 27.9 (8.2) |  |
| Range | 15.0 - 80.0 | 15.0 - 69.0 | 18.0 - 77.0 | 15.0 - 80.0 |  |
| Age (categories) |  |  |  |  | < 0.001^b^ |
| 18-24 | 78 (50.6%) | 721 (61.6%) | 4004 (38.5%) | 4803 (41.0%) |  |
| 25-29 | 29 (18.8%) | 221 (18.9%) | 2798 (26.9%) | 3048 (26.0%) |  |
| 30-39 | 31 (20.1%) | 120 (10.3%) | 2643 (25.4%) | 2794 (23.8%) |  |
| 40+ | 16 (10.4%) | 108 (9.2%) | 956 (9.2%) | 1080 (9.2%) |  |
| Education |  |  |  |  | < 0.001^b^ |
| Secondary or less | 15 (9.9%) | 135 (11.7%) | 251 (2.5%) | 401 (3.5%) |  |
| High school | 22 (14.5%) | 271 (23.5%) | 2254 (22.0%) | 2547 (22.1%) |  |
| Technical | 63 (41.4%) | 454 (39.4%) | 1030 (10.1%) | 1547 (13.4%) |  |
| Bachelor’s or above | 52 (34.2%) | 293 (25.4%) | 6696 (65.4%) | 7041 (61.0%) |  |
| Missing/Unknown | 2 | 17 | 170 | 189 |  |
| Sexual identity |  |  |  |  | < 0.001^b^ |
| Gay | 3 (2.0%) | 24 (2.1%) | 8744 (84.1%) | 8771 (74.9%) |  |
| Bisexual | 34 (22.2%) | 271 (23.4%) | 1531 (14.7%) | 1836 (15.7%) |  |
| Heterosexual | 116 (75.8%) | 859 (74.1%) | 50 (0.5%) | 1025 (8.8%) |  |
| Prefer not to answer | 0 (0.0%) | 2 (0.2%) | 28 (0.3%) | 30 (0.3%) |  |
| Don’t know | 0 (0.0%) | 3 (0.3%) | 42 (0.4%) | 45 (0.4%) |  |
| Missing/Unknown | 1 | 11 | 6 | 18 |  |
| Homeless |  |  |  |  | < 0.001^b^ |
| No | 137 (95.8%) | 1062 (95.3%) | 10352 (99.5%) | 11551 (99.1%) |  |
| Yes | 5 (3.5%) | 50 (4.5%) | 49 (0.5%) | 104 (0.9%) |  |
| Prefer not to answer | 1 (0.7%) | 2 (0.2%) | 0 (0.0%) | 3 (0.0%) |  |
| Don’t know | 0 (0.0%) | 0 (0.0%) | 0 (0.0%) | 0 (0.0%) |  |
| Missing/Unknown | 11 | 56 | 0 | 67 |  |
| Ever tested for HIV |  |  |  |  | < 0.001^b^ |
| No | 36 (23.4%) | 366 (31.3%) | 2819 (27.6%) | 3221 (27.9%) |  |
| Yes | 114 (74.0%) | 801 (68.5%) | 7393 (72.3%) | 8308 (71.9%) |  |
| Prefer not to answer | 2 (1.3%) | 1 (0.1%) | 11 (0.1%) | 14 (0.1%) |  |
| Don’t know | 2 (1.3%) | 1 (0.1%) | 9 (0.1%) | 12 (0.1%) |  |
| Missing/Unknown | 0 | 1 | 169 | 170 |  |
| HIV status |  |  |  |  | < 0.001^b^ |
| Negative | 106 (68.8%) | 743 (63.5%) | 5937 (57.1%) | 6786 (57.9%) |  |
| Positive | 4 (2.6%) | 49 (4.2%) | 1138 (10.9%) | 1191 (10.2%) |  |
| Don’t know | 44 (28.6%) | 378 (32.3%) | 3326 (32.0%) | 3748 (32.0%) |  |

^a^ Linear model ANOVA.

^b^ chi-square test.
