# Supplementary material for: Measurement of Sexual Behavior Stigma in Cisgender Mexican Sexual Minority Men: Contextual Considerations of Living in Mexico or the United States
Source: Arch Sex Behav. 2025 Jul 14;54(7):2599–610. doi: 10.1007/s10508-025-03184-5 (PMC12457474; doi:10.1007/s10508-025-03184-5)
Supplement: Supplementary file 2 [file 10508_2025_3184_MOESM2_ESM.docx]

**Supplementary Table 2.** Factor loadings of the sexual behavior stigma items and inter-factor correlations for a three-factor model of sexual behavior stigma from the three group CFA

|  | AMIS — Born in U.S. | | | AMIS — Born in Mexico | | | ESEH | | |
| --- | --- | --- | --- | --- | --- | --- | --- | --- | --- |
|  | Friends & Family | Healthcare | General | Friends & Family | Healthcare | General | Friends &  Family | Healthcare | General |
| 1. Have you ever felt excluded from family activities because you have sex with men? | 0.713 | 0 | 0 | 0.699 | 0 | 0 | 0.699 | 0 | 0 |
| 2. Have you ever felt that family members have made discriminatory remarks or gossiped about you because you have sex with men? | 0.615 | 0 | 0 | 0.734 | 0 | 0 | 0.734 | 0 | 0 |
| 3. Have you ever felt rejected by your friends because you have sex with men? | 0.513 | 0 | 0 | 0.404 | 0 | 0 | 0.404 | 0 | 0 |
| 4. Have you ever felt afraid to go to health-care services because you have sex with men? | 0 | 0.88 | 0 | 0 | 0.951 | 0 | 0 | 0.951 | 0 |
| 5. Have you ever avoided going to health-care services because you have sex with men? | 0 | 0.846 | 0 | 0 | 0.828 | 0 | 0 | 0.828 | 0 |
| 6. Have you ever heard health-care providers gossiping about you (talking about you) because you have sex with men? | 0 | 0 | 0.267 | 0 | 0 | 0.383 | 0 | 0 | 0.383 |
| 7. Have you ever felt that you were not treated well in a health center because you have sex with men? | 0 | 0 | 0.399 | 0 | 0 | 0.492 | 0 | 0 | 0.492 |
| 8. Have you ever felt that the police refused to protect you because you have sex with men? | 0 | 0 | 0.383 | 0 | 0 | 0.242 | 0 | 0 | 0.242 |
| 9. Have you ever felt scared to be in public places because you have sex with men? | 0 | 0 | 0.592 | 0 | 0 | 0.654 | 0 | 0 | 0.654 |
| 10. Have you ever been verbally harassed and felt it was because you have sex with men? | 0 | 0 | 0.627 | 0 | 0 | 0.725 | 0 | 0 | 0.725 |
| 11. Have you ever been blackmailed by someone because you have sex with men? | 0 | 0 | 0.344 | 0 | 0 | 0.205 | 0 | 0 | 0.205 |
| 12. Has someone ever physically hurt you (pushed, shoved, slapped, hit, kicked, choked or otherwise physically hurt you)? [AND] Do you believe any of these experiences of physical violence was/were related to the fact that you have sex with men? | 0 | 0 | 0.261 | 0 | 0 | 0.236 | 0 | 0 | 0.236 |
| 13. Have you ever been forced to have sex when you did not want to? (By forced, I mean physically forced, coerced to have sex, or penetrated with an object, when you did not want to). [AND] Do you believe any of these experiences of sexual violence were related to the fact that you have sex with men? | 0 | 0 | 0.11 | 0 | 0 | -0.029 | 0 | 0 | -0.029 |
| Factor correlations |  |  |  |  |  |  |  |  |  |
| Friends and Family | 1 | 0.384 | 0.772 | 1 | 0.348 | 0.833 | 1 | 0.348 | 0.833 |
| Healthcare | 0.38 | 1 | 0.399 | 0.348 | 1 | 0.479 | 0.348 | 1 | 0.479 |
| General | 0.772 | 0.399 | 1 | 0.833 | 0.479 | 1 | 0.833 | 0.479 | 1 |
