# Supplementary material for: Measurement of Sexual Behavior Stigma in Cisgender Mexican Sexual Minority Men: Contextual Considerations of Living in Mexico or the United States
Source: Arch Sex Behav. 2025 Jul 14;54(7):2599–610. doi: 10.1007/s10508-025-03184-5 (PMC12457474; doi:10.1007/s10508-025-03184-5)
Supplement: Supplementary file 3 [file 10508_2025_3184_MOESM3_ESM.docx]

**Supplementary Table 3.** Demographic characteristics of Mexican SMM across groups, AMIS (2018-2019) Born in Mexico or Born in the U.S., ESEH (2017): Comparison between complete cases and removed cases

|  | Complete Cases (N=11,725) | Removed Cases (N=5,114) | Total (N=16,839) | p value |
| --- | --- | --- | --- | --- |
| Age |  |  |  | 0.704 |
| Mean (SD) | 27.9 (8.2) | 27.9 (8.7) | 27.9 (8.4) |  |
| Range | 15.0 - 80.0 | 15.0 - 78.0 | 15.0 - 80.0 |  |
| Age (categories) |  |  |  | < 0.001 |
| 18-24 | 4803 (41.0%) | 2184 (42.7%) | 6987 (41.5%) |  |
| 25-29 | 3048 (26.0%) | 1235 (24.1%) | 4283 (25.4%) |  |
| 30-39 | 2794 (23.8%) | 1146 (22.4%) | 3940 (23.4%) |  |
| 40+ | 1080 (9.2%) | 549 (10.7%) | 1629 (9.7%) |  |
| Education |  |  |  | 0.036 |
| Secondary or less | 401 (3.5%) | 175 (3.5%) | 576 (3.5%) |  |
| High school | 2547 (22.1%) | 1157 (23.1%) | 3704 (22.4%) |  |
| Technical | 1547 (13.4%) | 593 (11.8%) | 2140 (12.9%) |  |
| Bachelor’s or above | 7041 (61.0%) | 3093 (61.6%) | 10134 (61.2%) |  |
| Missing/Unknown | 189 | 96 | 285 |  |
| Sexual identity |  |  |  | < 0.001 |
| Gay | 8771 (74.9%) | 3511 (68.8%) | 12282 (73.1%) |  |
| Bisexual | 1836 (15.7%) | 1089 (21.3%) | 2925 (17.4%) |  |
| Heterosexual | 1025 (8.8%) | 401 (7.9%) | 1426 (8.5%) |  |
| Prefer not to answer | 30 (0.3%) | 41 (0.8%) | 71 (0.4%) |  |
| Don’t know | 45 (0.4%) | 60 (1.2%) | 105 (0.6%) |  |
| N-Miss | 18 | 12 | 30 |  |
| Homeless |  |  |  | 0.182 |
| No | 11551 (99.1%) | 5009 (98.8%) | 16560 (99.0%) |  |
| Yes | 104 (0.9%) | 58 (1.1%) | 162 (1.0%) |  |
| Prefer not to answer | 3 (0.0%) | 2 (0.0%) | 5 (0.0%) |  |
| Don’t know | 0 (0.0%) | 1 (0.0%) | 1 (0.0%) |  |
| Missing/Unknown | 67 | 44 | 111 |  |
| Ever tested for HIV |  |  |  | < 0.001 |
|  |  |  |  |  |
| No | 3221 (27.9%) | 1628 (32.3%) | 4849 (29.2%) |  |
| Yes | 8308 (71.9%) | 3376 (66.9%) | 11684 (70.4%) |  |
| Prefer not to answer | 14 (0.1%) | 23 (0.5%) | 37 (0.2%) |  |
| Don’t know | 12 (0.1%) | 18 (0.4%) | 30 (0.2%) |  |
| Missing/Unknown | 170 | 69 | 239 |  |
| HIV status |  |  |  | < 0.001 |
| Negative | 6786 (57.9%) | 2755 (53.9%) | 9541 (56.7%) |  |
| Positive | 1191 (10.2%) | 480 (9.4%) | 1671 (9.9%) |  |
| Don’t know | 3748 (32.0%) | 1879 (36.7%) | 5627 (33.4%) |  |
