# Supplementary material for: Measurement of Sexual Behavior Stigma in Cisgender Mexican Sexual Minority Men: Contextual Considerations of Living in Mexico or the United States
Source: Arch Sex Behav. 2025 Jul 14;54(7):2599–610. doi: 10.1007/s10508-025-03184-5 (PMC12457474; doi:10.1007/s10508-025-03184-5)
Supplement: Supplementary file 4 [file 10508_2025_3184_MOESM4_ESM.docx]

**Supplementary Table 4.** Comparison of endorsement of sexual behavior stigma items among Mexican SMM: Complete cases vs. excluded cases

| Item (stigma type) | Complete (N=11,725) | Missing (N=5,114) | Total (N=16,839) | *p* value ^a^ |
| --- | --- | --- | --- | --- |
| 1. Have you ever felt excluded from family activities because you have sex with men? (perceived) |  |  |  | < 0.001 |
| N-Miss | 0 | 1813 | 1813 |  |
| No | 9015 (76.9%) | 2372 (71.9%) | 11387 (75.8%) |  |
| Yes | 2710 (23.1%) | 929 (28.1%) | 3639 (24.2%) |  |
| 2. Have you ever felt that family members have made discriminatory remarks or gossiped about you because you have sex with men? (perceived) |  |  |  | < 0.001 |
| N-Miss | 0 | 1891 | 1891 |  |
| No | 6544 (55.8%) | 1473 (45.7%) | 8017 (53.6%) |  |
| Yes | 5181 (44.2%) | 1750 (54.3%) | 6931 (46.4%) |  |
| 3. Have you ever felt rejected by your friends because you have sex with men? (perceived) |  |  |  | < 0.001 |
| N-Miss | 0 | 1088 | 1088 |  |
| No | 9425 (80.4%) | 3063 (76.1%) | 12488 (79.3%) |  |
| Yes | 2300 (19.6%) | 963 (23.9%) | 3263 (20.7%) |  |
| 4. Have you ever felt afraid to go to healthcare services because you worry someone may learn you have sex with men? (anticipated) |  |  |  | < 0.001 |
| N-Miss | 0 | 680 | 680 |  |
| No | 9342 (79.7%) | 2991 (67.5%) | 12333 (76.3%) |  |
| Yes | 2383 (20.3%) | 1443 (32.5%) | 3826 (23.7%) |  |
| 5. Have you ever avoided going to healthcare services because you worry someone may learn you have sex with men? (anticipated) |  |  |  | < 0.001 |
| N-Miss | 0 | 627 | 627 |  |
| No | 10046 (85.7%) | 3438 (76.6%) | 13484 (83.2%) |  |
| Yes | 1679 (14.3%) | 1049 (23.4%) | 2728 (16.8%) |  |
| 6. Have you ever felt that you were not treated well in a health center because someone knew that you have sex with men? (perceived) |  |  |  | 0.344 |
| N-Miss | 0 | 1430 | 1430 |  |
| No | 10814 (92.2%) | 3380 (91.7%) | 14194 (92.1%) |  |
| Yes | 911 (7.8%) | 304 (8.3%) | 1215 (7.9%) |  |
| 7. Have you ever heard healthcare providers gossiping about you (talking about you) because you have sex with men? (enacted) |  |  |  | < 0.001 |
| N-Miss | 0 | 1512 | 1512 |  |
| No | 10751 (91.7%) | 3234 (89.8%) | 13985 (91.2%) |  |
| Yes | 974 (8.3%) | 368 (10.2%) | 1342 (8.8%) |  |
| 8. Have you ever felt that the police refused to protect you because you have sex with men? (perceived) |  |  |  | 0.006 |
| N-Miss | 0 | 2300 | 2300 |  |
| No | 9912 (84.5%) | 2319 (82.4%) | 12231 (84.1%) |  |
| Yes | 1813 (15.5%) | 495 (17.6%) | 2308 (15.9%) |  |
| 9. Have you ever felt scared to be in public places because you have sex with men? (perceived) |  |  |  | < 0.001 |
| N-Miss | 0 | 959 | 959 |  |
| No | 8792 (75.0%) | 2831 (68.1%) | 11623 (73.2%) |  |
| Yes | 2933 (25.0%) | 1324 (31.9%) | 4257 (26.8%) |  |
| 10. Have you ever been verbally harassed and felt it was because you have sex with men? (enacted) |  |  |  | < 0.001 |
| N-Miss | 0 | 814 | 814 |  |
| No | 6319 (53.9%) | 2096 (48.7%) | 8415 (52.5%) |  |
| Yes | 5406 (46.1%) | 2204 (51.3%) | 7610 (47.5%) |  |
| 11. Have you ever been blackmailed by someone because you have sex with men? (enacted) |  |  |  | < 0.001 |
| N-Miss | 0 | 636 | 636 |  |
| No | 9811 (83.7%) | 3638 (81.2%) | 13449 (83.0%) |  |
| Yes | 1914 (16.3%) | 840 (18.8%) | 2754 (17.0%) |  |
| 12. Has someone ever physically hurt you (pushed, shoved, slapped, hit, kicked, choked or otherwise physically hurt you)? [AND] Do you believe any of these experiences of physical violence was/were related to the fact that you have sex with men? (enacted) |  |  |  | 0.024 |
| N-Miss | 0 | 255 | 255 |  |
| No | 10116 (86.3%) | 4256 (87.6%) | 14372 (86.7%) |  |
| Yes | 1609 (13.7%) | 603 (12.4%) | 2212 (13.3%) |  |
| 13. Have you ever been forced to have sex when you did not want to (by forced, I mean physically forced, coerced to have sex, or penetrated with an object, when you did not want to)? [AND] Do you believe any of these experiences of sexual violence were related to the fact that you have sex with men? (enacted) |  |  |  | 0.13 |
| N-Miss | 0 | 282 | 282 |  |
| No | 10957 (93.4%) | 4546 (94.1%) | 15503 (93.6%) |  |
| Yes | 768 (6.6%) | 286 (5.9%) | 1054 (6.4%) |  |

^a^ chi-square test.
