# Supplementary material for: Measurement of Sexual Behavior Stigma in Cisgender Mexican Sexual Minority Men: Contextual Considerations of Living in Mexico or the United States
Source: Arch Sex Behav. 2025 Jul 14;54(7):2599–610. doi: 10.1007/s10508-025-03184-5 (PMC12457474; doi:10.1007/s10508-025-03184-5)
Supplement: Supplementary file 5 [file 10508_2025_3184_MOESM5_ESM.docx]

**Supplementary Table 5.1. Measurement invariance testing, AMIS and ESEH datasets (Two groups*): with Pairwise Deletion**

| Model | X2 | df | p-value | RMSEA | 90% CI lower | 90% CI upper | CFI | TLI | SRMR |
| --- | --- | --- | --- | --- | --- | --- | --- | --- | --- |
| AMIS model | 267.899 | 62 | 0 | 0.043 | 0.038 | 0.048 | 0.965 | 0.956 | 0.059 |
| ESEH model | 1573.313 | 62 | 0 | 0.04 | 0.039 | 0.042 | 0.97 | 0.963 | 0.043 |
| Configural model | 1841.212 | 124 | 0 | 0.041 | 0.039 | 0.042 | 0.97 | 0.962 | 0.042 |
| Metric model | 2689.525 | 134 | 0 | 0.048 | 0.046 | 0.049 | 0.955 | 0.948 | 0.046 |
| Adjusted Metric model | 1905.51 | 128 | 0 | 0.041 | 0.039 | 0.042 | 0.969 | 0.962 | 0.042 |
| Adjusted Scalar model | 1908.269 | 133 | 0 | 0.04 | 0.038 | 0.042 | 0.969 | 0.963 | 0.042 |

*Two groups included Mexican SMM from the ESEH data and Mexican SMM from the AMIS data

**Supplementary Table 5.2. Measurement invariance testing, AMIS and ESEH datasets (Three groups*): with Pairwise Deletion**

| Model | X2 | df | p-value | RMSEA | 90% CI lower | 90% CI upper | CFI | TLI | SRMR |
| --- | --- | --- | --- | --- | --- | --- | --- | --- | --- |
| ESEH model | 1573.313 | 62 | 0 | 0.04 | 0.039 | 0.042 | 0.97 | 0.963 | 0.043 |
| AMIS US model | 240.272 | 62 | 0 | 0.042 | 0.037 | 0.048 | 0.963 | 0.954 | 0.06 |
| AMIS Mexico model | 62.43 | 62 | 0.461 | 0.006 | 0 | 0.042 | 1 | 0.999 | 0.079 |
| Configural model | 1876.016 | 186 | 0 | 0.04 | 0.039 | 0.042 | 0.97 | 0.963 | 0.042 |
| Metric model | 2798.246 | 206 | 0 | 0.048 | 0.046 | 0.049 | 0.955 | 0.948 | 0.046 |
| Adjusted Metric model | 2025.114 | 198 | 0 | 0.041 | 0.039 | 0.042 | 0.968 | 0.962 | 0.043 |
| Adjusted Scalar model | 2082.671 | 212 | 0 | 0.04 | 0.038 | 0.041 | 0.967 | 0.964 | 0.043 |

*Three groups included (1) Mexican SMM from ESEH, (2) Mexican SMM born in U.S. from AMIS, (3) Mexican SMM born in Mexico from AMIS
